# Association Between ABO Blood Group System and COVID-19 Susceptibility in Wuhan

**DOI:** 10.3389/fcimb.2020.00404

**Published:** 2020-07-21

**Authors:** Qian Fan, Wei Zhang, Bo Li, De-Jia Li, Jian Zhang, Fang Zhao

**Affiliations:** ^1^Department of Cardiology, Zhongnan Hospital of Wuhan University, Wuhan University, Wuhan, China; ^2^The State Key Laboratory Breeding Base of Basic Science of Stomatology (Hubei-MOST) & Key Laboratory of Oral Biomedicine Ministry of Education, School and Hospital of Stomatology, Wuhan University, Wuhan, China; ^3^Department of Oral Radiology, School and Hospital of Stomatology, Wuhan University, Wuhan, China; ^4^Department of Preventive Health, School of Health Sciences, Wuhan University, Wuhan, China

**Keywords:** ABO blood group system, COVID-19, association analysis, female, lymphocyte count

## Abstract

**Background:** The ABO blood group system has been associated with multiple infectious diseases, including hepatitis B, dengue haemorrhagic fever and so on. Coronavirus disease 2019 (COVID-19) is a new respiratory infectious disease and the relationship between COVID-19 and ABO blood group system needs to be explored urgently.

**Methods:** A hospital-based case-control study was conducted at Zhongnan Hospital of Wuhan University from 1 January 2020 to 5 March 2020. A total of 105 COVID-19 cases and 103 controls were included. The blood group frequency was tested with the chi-square statistic, and odds ratios (ORs) with 95% confidence intervals (CIs) were calculated between cases and controls. In addition, according to gender, the studied population was divided into two subgroups, and we assessed the association between cases and controls by gender. Finally, considering lymphopenia as a feature of COVID-19, the relationship between the ABO blood group and the lymphocyte count was determined in case samples.

**Results:** The frequencies of blood types A, B, AB, and O were 42.8, 26.7, 8.57, and 21.9%, respectively, in the case group. Association analysis between the ABO blood group and COVID-19 indicated that there was a statistically significant difference for blood type A (*P* = 0.04, OR = 1.33, 95% CI = 1.02–1.73) but not for blood types B, AB or O (*P* = 0.48, OR = 0.90, 95% CI = 0.66–1.23; *P* = 0.61, OR = 0.88, 95% CI = 0.53–1.46; and *P* = 0.23, OR = 0.82, 95% CI = 0.58–1.15, respectively). An analysis stratified by gender revealed that the association was highly significant between blood type A in the female subgroup (*P* = 0.02, OR = 1.56, 95% CI = 1.08–2.27) but not in the male subgroup (*P* = 0.51, OR = 1.14, 95% CI = 0.78–1.67). The average level of lymphocyte count was the lowest with blood type A in patients, however, compared with other blood types, there was still no significant statistical difference.

**Conclusions:** Our findings provide epidemiological evidence that females with blood type A are susceptible to COVID-19. However, these research results need to be validated in future studies.

## Introduction

Coronavirus disease 2019 (COVID-19), also named novel coronavirus pneumonia (NCP), was first reported in Wuhan in December 2019 and then gradually spread throughout the country. By early March 2020, more than 80,000 people were infected, nearly 3,200 of whom died in China. The pneumonia outbreak has become a serious public health event. COVID-19 is caused by severe acute respiratory syndrome coronavirus-2 (SARS-CoV-2), which is a new member of the coronavirus family. There are currently 7 known coronaviruses that can infect humans, such as severe acute respiratory syndrome (SARS) coronavirus and Middle East respiratory syndrome (MERS) coronavirus. Based on current epidemiological investigations, the incubation period is 1–14 days and typically 3–7 days, but there are also cases in which an incubation period of over 14 days is reported (Wang et al., 2020; Wu and McGoogan, 2020). Individuals are contagious during the incubation period, and asymptomatic infection may also become the source of infection. Respiratory droplets and close contact are the major transmission routes. COVID-19 is clinically characterized by fever, fatigue, and dry cough. In severe cases, affected individuals can undergo acute respiratory distress syndrome, septic shock, and even death (Chan et al., 2020; Huang et al., 2020).

The ABO blood group is the most important blood group system in humans and includes 4 blood types, namely, A, AB, B, and O. The human ABO blood group is located on chromosome 9 (9q34.2) (Melzer et al., 2008; Wiggins et al., 2009). Many studies have found that the ABO blood group plays an important role in various human diseases, such as cardiovascular, oncological, and some infectious and non-infectious diseases (Wolpin et al., 2010; Chen et al., 2016). Meanwhile, the system can play a direct role in infection by serving as receptors or coreceptors for microorganisms, parasites, and viruses. Blood group antigens, also named human histo-blood group antigens (HBGAs), are one of the main antigens on the surface of human red blood cells. They represent polymorphic traits inherited among individuals and populations. Differences in blood group antigen expression can increase or decrease host susceptibility to many infections. In addition, many blood group antigens facilitate intracellular uptake, signal transduction, or adhesion through the organization of membrane microdomains and modify the innate immune response to infection (Behal et al., 2010; Singh et al., 2016; Chakrani et al., 2018; Liu et al., 2018).

The ABO blood group has been previously found to contribute to the risk of multiple infectious diseases in a series of studies. Mohammadali et al. reported that the presence of blood group O might significantly decrease the risk of hepatitis B, and the distribution of Rh in HBV-infected individuals was higher between Rh-positive donors (Mohammadali and Pourfathollah, 2014). Elnady et al. found that Rota-positive status for rotavirus gastroenteritis was significantly more prevalent among those with blood type A and significantly less prevalent among those with blood type B (Elnady et al., 2017). Another recent study carried out by Degarege et al. reported that malaria patients with blood group A had a higher risk of anemia than did those with O and non-A phenotypes (Degarege et al., 2012). Among patients infected with dengue virus, Murugananthan et al. found that patients with AB blood had a risk that was more than 2.5 times higher of developing dengue haemorrhagic fever than did those with other blood types (Murugananthan et al., 2018). In addition, a meta-analysis suggested that blood types A, B, and AB might not affect susceptibility to norovirus infection. However, those with blood type O appeared to be more susceptible to this infection (Liao et al., 2020). Because SARS-CoV-2 is a completely new virus, it is unclear whether the ABO blood groups affect individuals' susceptibility to COVID-19.

Hence, we performed a case-control study to explore the relationship between the ABO blood group and COVID-19 in Wuhan and further classified the populations according to gender. Additionally, lymphopenia is a common feature of patients with COVID-19 and might be a critical factor associated with the severity and mortality of the disease (Xu Z. et al., 2020). The association between ABO blood type and the count of lymphocyte was also investigated in cases.

## Methods

### Study Design and Data Source

A retrospective case-control association study was performed during the period from 1 January 2020 to 5 March 2020, with a total of 208 subjects (105 cases vs. 103 controls). All subjects were enrolled from Zhongnan Hospital of Wuhan University, which is a hospital designated for the treatment of patients with COVID-19.

All study individuals were subjected to demographics, clinical features, laboratory findings, reports, and chest CT scans. Demographics included age, gender, hypertension, diabetes, and heart disease. Clinical features involved disease manifestations such as fever, cough, dyspnoea, chest tightness, and diarrhea. Laboratory findings included white blood cell count, lymphocyte count, neutrophil ratio, lymphocyte ratio, blood type, and throat swab nucleic acid test results. All information was obtained and analyzed with the standard Excel program. Two doctors independently extracted the data of the eligible individuals, and the results were reviewed by a third investigator.

This study was reviewed and approved by the Medical Ethical Committee of Zhongnan Hospital of Wuhan University. Oral consent was obtained from patients.

### Case and Control Selection

The criterion for enrolment as a case was defined according to the Diagnosis and Treatment Scheme for New Coronavirus Pneumonia (Trial version 5, Trial version 6) issued by the General Office of National Health Commission of the People's Republic of China and the Office of State Administration of Traditional Chinese Medicine.

COVID-19 cases were diagnosed as “clinically diagnosed cases” or “confirmed cases” according to the above criteria. The specific diagnostic criteria for clinically diagnosed cases are as follows: (a) history of epidemiology: I Travel history or residence history in Wuhan and surrounding areas within 14 days prior to onset of the disease, or other cases reported in the community; II contact with patients from Wuhan and surrounding areas, or with fever or respiratory symptoms from the community prior to the onset of the disease, within 14 days prior to onset of the disease; III cluster disease; and IV. contact with a new type of coronavirus infection; (b) clinical manifestations: I fever and/or respiratory symptoms; II imaging features of the above pneumonia; and III normal or decreased total white blood cell count or decreased lymphocyte count at the early stage of onset; and (c) comprehensive evaluation by three COVID-19 consultation experts in the hospital. The specific diagnostic criterion for confirmed cases is as follows: COVID-19 nuclear acid test positive for viral nucleic acid by reverse transcription polymerase chain reaction real-time (RT-PCR) detection with specimens from the respiratory tract or blood samples.

The eligible control subjects were selected from individuals with the following characteristics: (1) gender- and age-matched; (2) no other history of respiratory infections, such as bacterial pneumonia, mycoplasma pneumonia, tuberculosis and other types of pneumonia; (3) no other infectious diseases, such as hepatitis B and AIDS; and (4) no severe liver and kidney dysfunction.

### Association Analysis

The association between different blood types and COVID-19 was performed in the selected population. According to gender, subgroups were stratified to assess whether there was a significant difference between blood type and the incidence of COVID-19. In addition, because lymphocyte decline was related to the severity of COVID-19, we performed a correlation analysis between blood group and lymphocyte count in the COVID-19 patients (Chen et al., 2020).

### Statistical Analysis

Statistical analysis was carried out using the Statistical Package for Social Sciences (SPSS) version 21.0. Independent sample *t*-tests were used for age, white blood cell count, lymphocyte count, neutrophil ratio, and lymphocyte ratio. A chi-square test was used for hypertension, diabetes, heart disease, tumor, liver disease, and kidney disease. The ABO blood group frequency in all populations and different gender subgroups was tested using chi-square tests and odds ratios (ORs) with 95% confidence intervals (CIs). Analysis of the association between the ABO blood group and the lymphocyte count was performed with analysis of variance (ANOVA) and a linear regression model. A *P* < 0.05 was considered significant.

## Results

### Distribution of the ABO Blood Group System

Table 1 illustrates the demographic, clinical, and laboratory characteristics of the study population. The present research consisted of 208 participants divided into two groups: the COVID-19 case group and the control group. Of the 105 patients with COVID-19, 55 were males and 50 were females. The age range of patients was 56.8 ± 18.3. The frequencies of blood types A, B, AB, and O were 42.8, 26.7, 8.57, and 21.9%, respectively. In the control group, 56 (54.4%) of the participants were males, and 47 (45.6%) were females. The age range of the control subjects was 54.0 ± 15.0. The distribution of the ABO blood group of the controls was 29.1% for A, 31.1% for B, 29.1% for O and 10.7% for AB.

**Table 1:** The clinical characteristics of the studied population.

**Characteristics**	**Case**	**Control**	***P***
Number of subjects	105	103	−
Age (years)	56.8 ± 18.3	54.0 ± 15.0	0.228
Gender (male %)	55 (52.4%)	56 (54.4%)	0.774
Hypertension (%)	36 (34.0%)	20 (19.4%)	0.019
Diabetes (%)	11 (10.5%)	9 (8.74%)	0.815
Heart disease (%)	18 (17.1%)	10 (9.71%)	0.155
Tumor (%)	5 (4.76%)	6 (5.83%)	0.767
Liver disease (%)	3 (2.86%)	0 (0.00%)	0.246
Kidney disease (%)	9 (8.57%)	2 (1.94%)	0.06
White blood cell count (10∧9/L)	6.94 ± 3.66	6.27 ± 1.75	0.091
Lymphocyte count (10∧9/L)	0.81 ± 0.47	1.64 ± 0.49	<0.001
Neutrophil ratio (%)	76.8 ± 13.6	62.3 ± 10.1	<0.001
Lymphocyte ratio (%)	14.4 ± 10.5	27.6 ± 9.37	<0.001

*The data are presented as the mean ± standard deviation or a percentage*.

### Association Between ABO Blood Group and COVID-19

As shown in Table 2, we performed a combined association analysis between ABO blood group and COVID-19, which showed a statistically significant difference in COVID-19 infection among those with blood type A (*P* = 0.04, OR = 1.33, 95% CI = 1.02–1.73) but not blood types B, AB or O (*P* = 0.48, OR = 0.90, 95% CI = 0.66–1.23; *P*=0.61, OR = 0.88, 95% CI = 0.53–1.46; and *P* = 0.23, OR = 0.82, 95% CI = 0.58–1.15, respectively).

**Table 2:** Association analysis of ABO blood type between COVID-19 cases and controls.

**Blood**	**Case (%)**	**Control (%)**	***χ^2^***	***P***	**OR (95% CI)**
**group**					
A	45 (42.8%)	30 (29.1%)	4.25	0.04	1.33 (1.02–1.73)
B	28 (26.7%)	32 (31.1%)	0.49	0.48	0.90 (0.66–1.23)
AB	9 (8.57%)	11 (10.7%)	0.27	0.61	0.88 (0.53–1.46)
O	23 (21.9%)	30 (29.1%)	1.43	0.23	0.82 (0.58–1.15)

*OR, odds ratio after adjustment; CI, confidence interval*.

### Stratified Analysis by Gender

An additional statistical analysis was performed by dividing the entire population into two subgroups by gender, as shown in Table 3. The male group comprises 111 subjects, and the female group includes 97 individuals. The association analysis revealed a significant relation between blood type A and COVID-19 in the female subgroup (*P* = 0.02, OR = 1.56, 95% CI = 1.08–2.27) but not in the male subgroup (*P* = 0.51, OR = 1.14, 95% CI = 0.78–1.67).

**Table 3:** Gender-stratified analysis of ABO blood type and COVID-19 cases.

**Blood group**	**Male**		***χ^2^***	***P***	**OR (95% CI)**	**Female**		***χ^2^***	***P***	**OR (95% CI)**
	**Case**	**Control**				**Case**	**Control**			
A	21	18	0.44	0.51	1.14 (0.78–1.67)	24	12	5.24	0.02	1.56 (1.08–2.27)
B	17	19	0.12	0.73	0.93 (0.62–1.41)	11	13	0.42	0.52	0.85 (0.53–1.39)
AB	6	5	0.12	0.73	1.13 (0.63–1.98)	3	6	1.32	0.25	0.62 (0.24–1.61)
O	11	14	0.40	0.53	0.86 (0.53–1.40)	12	16	1.19	0.28	0.78 (0.48–1.26)

*OR, odds ratio after adjustment; CI, confidence interval*.

In addition, blood types B, AB, and O were not significantly associated in either male or female subgroups (*P* > 0.05).

### Association Between Lymphocyte Count and COVID-19

**Figure 1:**
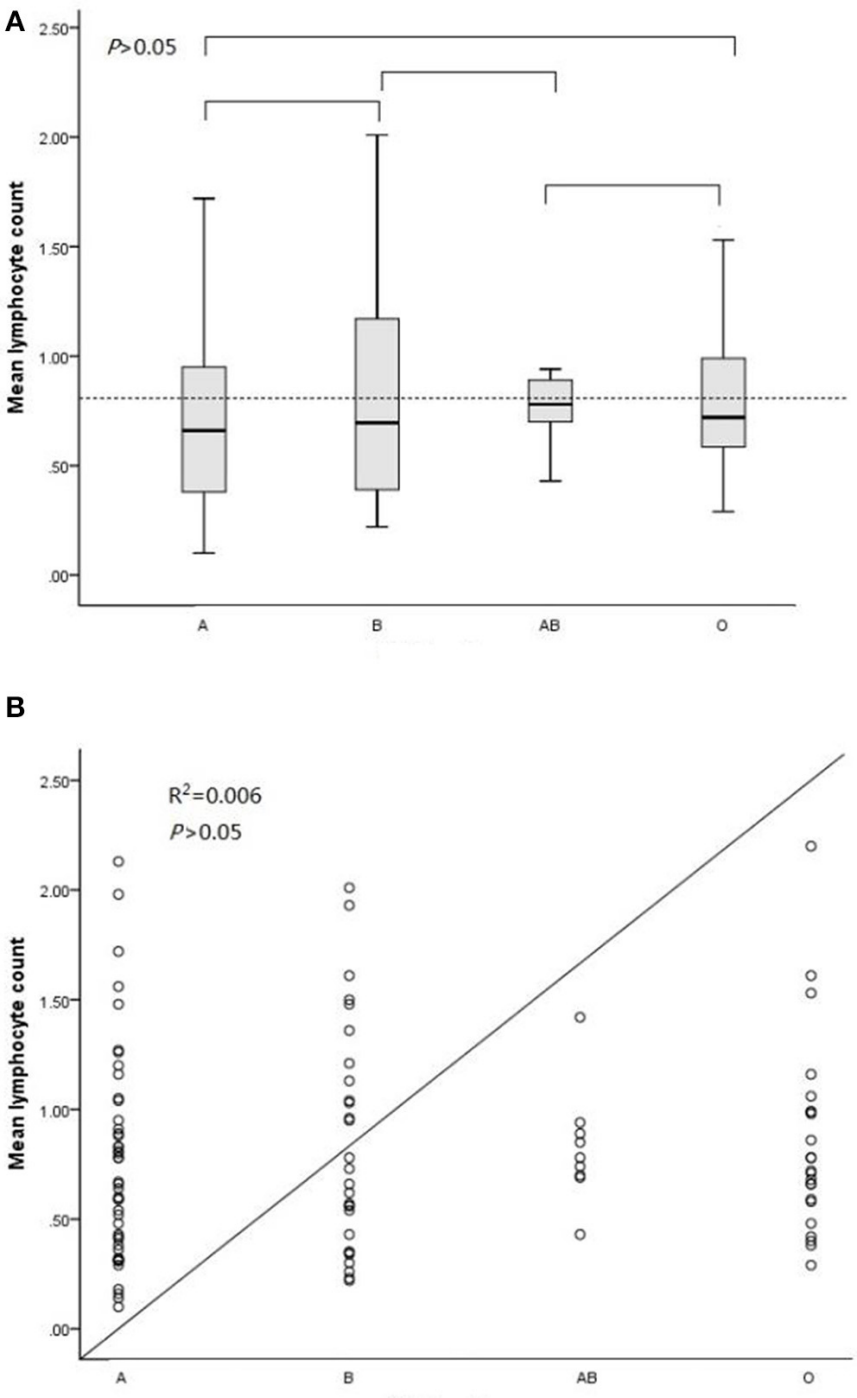
ABO blood type and lymphocyte count level analysis in the case group. **(A)** Comparisons among every pair of the four blood types via ANOVA. **(B)** Association analysis between the lymphocyte count and ABO blood type in a linear regression model.

As illustrated in Table 4 and Figure 1, the average lymphocyte count levels of individuals with blood type A were lower than those of individuals with blood types B, AB, and O in the case group (0.76^*^10^9^/L, 0.85^*^10^9^/L, 0.83^*^10^9^/L and 0.85^*^10^9^/L, respectively).

**Table 4:** Association analysis between the lymphocyte count and ABO blood type in COVID-19 cases.

**Blood grouping**	***n***	**Mean ± SD (10∧9/L)**	**95% CI**	**F**	***P***
A	45	0.76 ± 0.48	0.61–0.90		
B	28	0.85 ± 0.52	0.65–1.05		
AB	9	0.83 ± 0.27	0.62–1.03	0.30	0.83
O	23	0.85 ± 0.45	0.60–1.04		

*SD, standard deviation; CI, confidence intervals*.

Unfortunately, statistical analysis showed that blood type A was not significantly associated with lymphocyte count levels in case subjects (*P* = 0.83, F = 0.30).

## Discussion

Of the human blood group systems, the ABO blood group is widely used in clinical practice. As some of the important antigens, HBGAs are complex carbohydrate molecules with specific oligosaccharide sequences expressed on the surface of red blood cell membranes. These antigens are also highly expressed on a large number of human cells and tissues, including epithelia, platelets, vascular endothelia and neurons (Storry and Olsson, 2009; Liumbruno and Franchini, 2013; Heggelund et al., 2017; Kazi et al., 2017). HBGAs have been postulated to modify the spread of pathogens through the action of natural antibodies and complements (Neil et al., 2005; Ewald and Sumner, 2018). ABO antibodies are part of the innate immune system against some parasites, bacteria and enveloped viruses, and HBGAs are important as receptors for immune and inflammatory responses (Cooling, 2015; Jing et al., 2020). Meanwhile, this system is often used as a genetic marker in the human genome, generated by a polymorphic glycosyl-transferase encoded by 2 dominant active and a recessive inactive alleles. The association between ABO blood groups and infectious and non-infectious diseases has been widely explored (Groot et al., 2020).

In the current study, we aimed to evaluate the contribution of the ABO blood group to COVID-19 susceptibility in Wuhan by employing a case-control association analysis. Our present results demonstrated that there was a significant association between the A blood group and COVID-19, such that females (but not males) with blood type A were more susceptible to COVID-19 infection. Compared with other patients, female patients with blood type A had a relative risk of 1.33 for coronavirus infection. Xiong et al. recently also found that women show different characteristics from men in the transmission of COVID-19 (Xiong et al., 2020). We speculate that this result may be related to the different anatomic structures, estrogen levels, immune systems and genetic backgrounds of men and women. We further investigated the possible association between ABO blood group and lymphocyte count, the latter was considered as one of the index to evaluate the severity of COVID-19. Although statistical analysis showed no significant difference in ABO blood group and lymphocyte counts, our study found that the decreased lymphocyte counts in patients with blood type A were lower than those in patients with other blood types. The possible explanation for this finding may be related to the small sample size.

In fact, a number of epidemiological studies had also been conducted. For instance, the study of Li et al. reported that the proportion of blood type A in patients infected with SARS-CoV-2 was significantly higher than that in healthy controls (0.38 vs. 0.32%, *P* < 0.001), while the proportion of blood type O in SARS-CoV-2 infected patients was significantly lower than in healthy controls (0.26 vs. 0.34%, *P* < 0.001) (Li et al., 2020). In another study, Zhao et al. also showed that blood type A was associated with an increased risk of SARS-CoV-2 infection, whereas blood type O was associated with a decreased risk (Gerard et al., 2020; Zhao et al., 2020). The main finding of our study was consistent with the above analysis by Li et al. and Zhao et al., but slightly different. In our cases, the relationship between ABO blood type and the count of lymphocyte was further investigated, due to the importance of lymphocyte count in the evaluation of severity of COVID-19.

As with COVID-19, SARS is also a serious respiratory infectious disease. Nevertheless, ABO blood group-associated susceptibility to SARS is different from the corresponding susceptibility to COVID-19. In 2005, Cheng et al. found that individuals with blood type O had a reduced susceptibility to SARS infection in the Hong Kong population. Variable binding affinity to differing ABH substances present in gut epithelial cells may be the cause of the above phenomenon (Cheng et al., 2005).

SARS-CoV-2 belongs to lineage B betacoronavirus and shares high sequence identity with that of bat or human severe acute respiratory syndrome coronavirus-related coronavirus (SARSr-CoV) (Tian et al., 2020). The structural analysis of SARS-CoV-2 contains two important viral proteins, the nucleocapsid and the spike (S) proteins. S proteins of coronaviruses are large transmembrane heavily N-glycosylated proteins that mediate association with a cell surface receptor. SARS-CoV-2 makes use of the S protein to gain entry into the host (Li et al., 2006; Wrapp et al., 2020). Angiotensin-converting enzyme 2 (ACE2) is the main host cell receptor of SARS-CoV-2 and plays a crucial role in the entry of the virus into the cell to cause the final infection (Cao et al., 2020; Wu, 2020; Xu H. et al., 2020). The relationship between natural antibodies of the ABO blood system and the ACE2 interaction has been experimentally investigated. In 2008, Guillon et al. observed that S protein/ACE2-dependent adhesion of special Chinese hamster ovary cells to an ACE2-expressing cell line could be specifically inhibited by either monoclonal or human natural anti-A antibodies. Their findings indicated that anti-A antibodies may block the interaction between the SARS coronavirus and its receptor-ACE2, thereby providing protection (Guillon et al., 2008). This is consistent with our findings, suggesting that those with blood type A may be more susceptible to viral infection.

Meanwhile, several drawbacks existed in our study. First, Due to the limited sample size of COVID-19 in the early stages, the sample size included in our research is not very large. Second, regional selection bias needs to be considered. Third, other potential diseases may affect the research results. Finally, some of the control individuals might develop COVID-19 in the future.

In conclusion, female patients with blood type A are susceptible to COVID-19 in Wuhan after gender stratification. However, more studies are necessary to confirm these findings in a larger sample and among individuals of different ethnicities. The underlying mechanism between the ABO blood groups and ACE2 needs to be further explored.

## Data Availability Statement

All datasets generated for this study are included in the article/supplementary material.

## Ethics Statement

The studies involving human participants were reviewed and approved by Medical Ethics Committee, Zhongnan Hospital of Wuhan University. The patients/participants provided their written informed consent to participate in this study. Written informed consent was obtained from the individual(s) for the publication of any potentially identifiable images or data included in this article.

## Author Contributions

FZ and QF had full access to all the data in the study and takes responsibility for the integrity of the data and the accuracy of the data analysis. QF, D-JL, and JZ performed statistical analysis. All authors contributed to the article and approved the submitted version.

## Conflict of Interest

The authors declare that the research was conducted in the absence of any commercial or financial relationships that could be construed as a potential conflict of interest.
